# Daily Activity Lifelogs of People With Heart Failure: Observational Study

**DOI:** 10.2196/51248

**Published:** 2024-02-21

**Authors:** Teketo Kassaw Tegegne, Ly-Duyen Tran, Rebecca Nourse, Cathal Gurrin, Ralph Maddison

**Affiliations:** 1 Deakin University Burwood Australia; 2 Dublin City University Dublin Ireland

**Keywords:** heart failure, self-management, lifelogs, daily activity, wearable camera, E-Myscéal, activities of daily living, ADL, intervention, self-report method, wearable, chronic condition

## Abstract

**Background:**

Globally, heart failure (HF) affects more than 64 million people, and attempts to reduce its social and economic burden are a public health priority. Interventions to support people with HF to self-manage have been shown to reduce hospitalizations, improve quality of life, and reduce mortality rates. Understanding how people self-manage is imperative to improve future interventions; however, most approaches to date, have used self-report methods to achieve this. Wearable cameras provide a unique tool to understand the lived experiences of people with HF and the daily activities they undertake, which could lead to more effective interventions. However, their potential for understanding chronic conditions such as HF is unclear.

**Objective:**

This study aimed to determine the potential utility of wearable cameras to better understand the activities of daily living in people living with HF.

**Methods:**

The “Seeing is Believing (SIB)” study involved 30 patients with HF who wore wearable cameras for a maximum of 30 days. We used the E-Myscéal web-based lifelog retrieval system to process and analyze the wearable camera image data set. Search terms for 7 daily activities (physical activity, gardening, shopping, screen time, drinking, eating, and medication intake) were developed and used for image retrieval. Sensitivity analysis was conducted to compare the number of images retrieved using different search terms. Temporal patterns in daily activities were examined, and differences before and after hospitalization were assessed.

**Results:**

E-Myscéal exhibited sensitivity to specific search terms, leading to significant variations in the number of images retrieved for each activity. The highest number of images returned were related to eating and drinking, with fewer images for physical activity, screen time, and taking medication. The majority of captured activities occurred before midday. Notably, temporal differences in daily activity patterns were observed for participants hospitalized during this study. The number of medication images increased after hospital discharge, while screen time images decreased.

**Conclusions:**

Wearable cameras offer valuable insights into daily activities and self-management in people living with HF. E-Myscéal efficiently retrieves relevant images, but search term sensitivity underscores the need for careful selection.

## Introduction

Heart failure (HF) refers to when the heart does not work as well as it should in pumping blood and oxygen around the body [1]. HF is one of the most common chronic conditions, affecting 64 million people worldwide [2]. Despite advancements in medical treatment, people diagnosed with HF remain at an increased risk of hospitalization, hospital readmission, and premature death [3]. People with HF are encouraged to engage in a range of daily self-care activities to help manage their condition and prevent deterioration of their condition, collectively referred to as self-management. Those strategies typically include taking prescribed medication, making lifestyle changes, self-weighing, and monitoring signs and symptoms, and responding accordingly [4]. Previous meta-analyses of interventions to support self-management have reported significant benefits for people living with HF, including reduced hospitalizations [5-7], improved quality of life, and reduced all-cause mortality [5,7]. Moreover, self-management empowers people living with HF to take an active role in their care and facilitate effective management of their condition [4].

Understanding what behaviors people with HF engage in, and when, is important to understand how to support people with HF to better manage their condition. However, previous research has often relied on self-report methods to achieve this; self-report is subject to recall and social desirability biases, and memory impairment [8-11]. Digital technologies, such as wearable cameras, can provide valuable insights into health management behaviors, including contextual information such as location, time of day, and setting. Further, wearable cameras also offer a unique tool for researchers and health care providers to gain a better understanding of people’s lived experiences, which could lead to more tailored and effective interventions and treatments [12].

However, using wearable cameras generates large data sets, requiring the retrieval, processing, and analysis of relevant images for practical utility. This involves assigning semantic contexts like visual descriptions, time, and location [13,14]. Various computer vision models are employed, such as object detection, activity recognition, and optical character recognition, in addition to embedding models [13,15-17]. Retrieval systems incorporate techniques such as query enhancement, visual similarity search, and temporal search [13,16]. Previous studies [18-20], including our own [21], have manually reviewed camera images, which is time-consuming. In this study, we used the E-Myscéal system [22] for efficient retrieval and review of relevant images depicting the daily activities of people living with HF.

Daily activities significantly impact the health and well-being of people with HF, and a balanced approach to activities such as physical activity, screen time, eating, drinking, and medication intake is crucial for effective symptom management and improved quality of life [23-25]. Thus, the overall aim of this study was to determine the utility of wearable cameras to better understand activities of daily living (ADLs) in people living with HF. This study focused on 7 daily activities: physical activity, gardening, shopping, screen time, drinking, eating, and medication intake behaviors. This study also aimed to evaluate the sensitivity of an image-processing software tool for identifying these activities.

## Methods

### Study Design

The Seeing is Believing (SIB) study was a large prospective observational pilot study that evaluated the feasibility and acceptability of using wearable cameras and point-of-care testing for self-care in patients with HF [21,26]. As a pilot study, no formal power calculations were performed to determine the optimum sample size. It involved 30 patients with HF in Melbourne, Victoria, Australia, who wore wearable cameras for a month and conducted regular self-assessments. However, in this study, we analyzed the wearable camera image data.

### Study Population, Recruitment, and Setting

Participants, aged 18 years or older, were recruited from a single-center HF outpatient clinic in Melbourne if they had a documented HF diagnosis, had previous HF hospitalization, were on maximum tolerated medication, and were able to read and understand English. Exclusion criteria included severe HF symptoms (New York Heart Association class IV), advanced malignancy, cognitive impairment, and end-of-life care. Recruitment occurred during outpatient HF clinic visits by a cardiologist or researcher, followed by screening for eligibility by a Deakin University researcher. Eligible individuals underwent a baseline assessment and provided written consent.

### Study Procedures

Participants were asked to attach the “Narrative Clip” wearable camera to their shirt or blouse during waking hours for a maximum of 30 days. The camera captured images every 30 seconds, resulting in a data set of approximately 2.2 million images. Baseline assessment included medical history, demographics, and physical measurements. A research assistant collected point-of-care test data, hospitalization information, and other variables twice a week during the 30-day study [26]. At the end of this study, participants underwent a brief interview about their camera usage experience. In the original SIB study, 10 individuals were hospitalized [26].

### Wearable Camera Image-Processing and Retrieval

The wearable camera image data were processed and retrieved using the E-Myscéal web-based lifelog retrieval system. The E-Myscéal system uses deep learning algorithms to create embeddings (vector representations) of various lifelog data types such as images, text, and audio, enabling intuitive and web-based cross-media querying [22]. E-Myscéal uses the CLIP model to retrieve images similar in content to descriptive textual search terms, allowing users to search through lifelog data with great flexibility [27]. E-Myscéal was ranked as the top retrieval system at the Lifelog Search Challenge (LSC’22 challenge), outperforming others in terms of finding the highest number of relevant items in the shortest time across various retrieval tasks [28].

**Figure 1:**
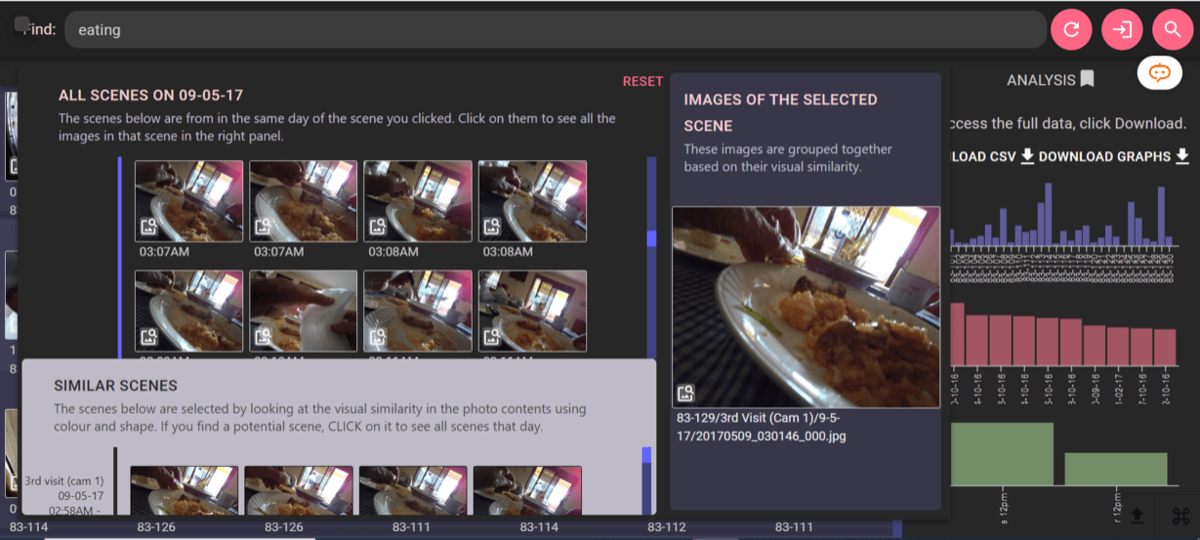
E-Myscéal user interface and event view window. Cam: camera.

With E-Myscéal, users can enter any search terms, leading to a list of related images along with date or time and location metadata [22]. For instance, when “eating” was used as a search term, E-Myscéal retrieved all food-related camera images, enabling researchers to assess the frequency of the wearer’s food consumption per day or over multiple days (see Figure 1). The system’s flexibility allows researchers to query data sets for any desired search terms, making it useful for various applications [22]. A demonstration of this is evident in the grouping of images within a *scene*, showcased in the right section of the pop-up within Figure 1.

Before applying the E-Myscéal system to our wearable camera data, we evaluated its capability to detect ADLs using a publicly available data set [29]. The results indicated a high precision in detecting ADLs such as physical activity, screen time, and shopping, with precision ranging from 0.8 to 1.0 [28]. Given these results, we decided to use the system with our data set. Detailed information on how E-Myscéal determined image counts can be found in Multimedia Appendix 1.

### Search Terms

Wearable camera images from the SIB study were uploaded to the E-Myscéal system [22]. Based on expert knowledge we developed search terms for each of the 7 daily activities (physical activity, gardening, shopping, screen time, drinking, eating, and taking medication), which were entered into E-Myscéal. Different search terms were used for each activity to identify the most appropriate ones for use. For example, for physical activity, search terms included “running,” “walking,” “yoga,” or “exercise,” which are common terms used to describe physical activity. Similarly, for gardening, search terms included “horticulture,” “cultivation,” “planting,” or “watering,” which are commonly used terms in gardening. This study assessed the relevance of search terms for each activity by analyzing the number and relevance of images returned.

### Data Analysis

We performed a sensitivity analysis using the Wilcoxon test to evaluate E-Myscéal’s effectiveness in retrieving relevant images with different search terms. The analysis involved comparing the number of images retrieved for 7 daily activities, both with and without outliers. For each activity, we tested the following search terms: physical activity (physical activity vs exercise), gardening (gardening vs horticulture), shopping (shopping vs retail therapy), screen time (screen time vs screen viewing), taking medication (taking medication vs medication intake), eating (food intake vs food consumption), and drinking (fluid intake vs hydrating). We assessed the normality assumption of each search term using the Shapiro-Wilk test and then used the Wilcoxon test to examine significant differences in the number of images retrieved for both searches (with and without outliers).

Following the sensitivity analysis, descriptive statistics were performed for ADLs using the most sensitive search terms. The results are presented in tables and figure. Furthermore, we used the Wilcoxon test to determine if there were significant temporal differences in daily activity patterns (ie, before and after midday). Further, we examined the ADLs of the 10 participants who were hospitalized during the 30-day study period. We compared their ADLs before they were admitted to the hospital and after they were discharged and returned home.

### Ethical Considerations

Ethical approval was granted by the Deakin University Human Research Ethics Committee (HREC/16/MH/55) and the Western Health Human Research Ethics Committee (2016.071). Participants were provided with study information through an information sheet and verbal explanation, with the option to withdraw at any time while retaining data collected to that point. All participants had an opportunity to ask questions about this study before they gave written informed consent. Participants could delete images from their wearable camera during this study. Upon completion, participants received an Aus $40 (US $28) voucher.

## Results

### Characteristics of Study Participants

In total, 30 adults (18 men) with HF agreed to participate and wore the wearable camera for up to 30 days. The median age of participants was 84 years, with a range of 47-96 years. Out of 30 participants, 20 were in New York Heart Association class III, and 10 were in class II. Among the 30 participants, 18 were diagnosed with HF within 5 years, and 10 were readmitted to the hospital due to HF exacerbation. For additional sample details, please refer to our previous publications [21,26]. No serious adverse events were reported with the use of the wearable camera.

### E-Myscéal Sensitivity to Search Terms

For each search term, the E-Myscéal system yielded varying numbers of images for each daily activity (Table 1). For example, the difference in the number of images for each daily activity ranged from 362 for screen time to 7015 for gardening. The Wilcoxon test confirmed a significant difference in the number of images retrieved using these search terms (Table 1). Based on the sensitivity analysis, the final search terms selected for each daily activity were as follows: physical activity, gardening, retail therapy, screen viewing, fluid intake, food intake, and taking medication.

**Table 1:** Sensitivity of the E-Myscéal system to search terms related to daily activities in a wearable camera pilot study on people with HF^a^.

Daily activities and search terms		Images retrieved, n	Wilcoxon test *P* value
**Physical activity**			*>*.05
	Physical activity	12,575	
	Exercise	11,083	
**Screen time**			*>*.05
	Screen viewing	16,968	
	Screen time	16,606	
**Taking medication**			*<*.05
	Taking medication	21,750	
	Medication intake	16,985	
**Shopping**			*<*.05
	Retail therapy	23,318	
	Shopping	21,221	
**Drinking**			*>*.05
	Fluid intake	23,773	
	Hydrating	19,020	
**Gardening**			*<*.05
	Gardening	25,585	
	Horticulture	18,570	
**Eating**			*>*.05
	Food intake	26,812	
	Food consumption	24,672	

^a^HF: heart failure.

### Activity Identification

For each of the 7 daily activities, the number of images returned ranged from 12,575 to 26,812. Eating had the highest number of images returned at 26,812, followed by gardening with 25,583 images, drinking with 23,773 images, and shopping with 23,318 images (Table 2).

**Table 2:** Daily activities of people living with HF^a^ in a wearable camera pilot study (N=30).

Daily activity	Images retrieved
Physical activity	12,575
Screen time	16,968
Taking medication	21,750
Shopping	23,318
Drinking	23,773
Gardening	25,583
Eating	26,812

^a^HF: heart failure.

### Activity Patterns During the Day

Most of the activities were observed before midday (Table 3). For example, 17,980 (67.1%) of images returned using search terms related to “eating” activity were captured before midday. Similarly, 17,065 (66.7%) gardening images, 15,245 (65.4%) shopping, and 15,219 (64%) drinking images were captured before midday. Additionally, there were statistically significant differences between daily activity patterns pre- and postmidday.

**Table 3:** Daily activities of people living with HF^a^ based on time of day in a wearable camera pilot study (N=30).

Daily activities	Before midday, n (%)	After midday, n (%)
Physical activity	7572 (60.2)	5003 (39.8)
Screen time	10,383 (61.2)	6585 (38.8)
Taking medication	13,299 (61.1)	8451 (38.9)
Shopping	15,245 (65.4)	8073 (34.6)
Drinking	15,219 (64)	8554 (36)
Gardening	17,065 (66.7)	8518 (33.3)
Eating	17,980 (67.1)	8832 (32.9)

^a^HF: heart failure.

### Activity Patterns Before and After Hospitalization

For the 10 participants that were hospitalized, there were marked differences in the percentage of ADLs images captured by a wearable camera pre- and posthospitalization, except for physical activity (Table 4). To illustrate, a total of 10,144 medication images were identified using E-Myscéal. Of those, 3827 (37.7%) were observed before hospital admission, while 6317 (62.3%) were observed posthospital discharge. In contrast, there was a decrease in screen time images after hospital discharge 946 (32.1%), compared to before admission 2003 (67.9%).

**Table 4:** Activities of daily living in patients with HF^a^ before and after hospitalization in a wearable camera pilot study (N=10).

Daily activities	Before hospital admission, n (%)	After hospital discharge, n (%)
Physical activity	2709 (50.5)	2659 (49.5)
Screen time	2003 (67.9)	946 (32.1)
Taking medication	3827 (37.7)	6317 (62.3)
Shopping	4161 (42.5)	5633 (57.5)
Drinking	3958 (35.4)	7211 (64.6)
Gardening	3142 (23.8)	10,061 (76.2)
Eating	5516 (40.7)	8038 (59.3)

^a^HF: heart failure.

## Discussion

### Principal Findings

This study aimed to determine the potential utility of wearable cameras to better understand ADLs in people living with HF. Overall, we showed that wearable cameras can be used to capture specific daily activities, which could be used to help identify areas where interventions may be targeted to improve people’s overall health and well-being. The E-Myscéal search engine was critical to the potential utility of this technology by rapidly retrieving images of relevance. Beyond, these general observations, specific issues are discussed below.

Typically, the use of wearable cameras results in large data sets, which means we have to retrieve, process, and analyze images of relevance for these data to be useful. The most common approach to organizing, retrieving, and analyzing data from wearable cameras involves assigning semantic contexts to images, like visual descriptions, time, and location [13,14]. Various computer vision models are employed to extract visual information from the images, including object detection, activity recognition, optical character recognition [13,15], and embedding models [16,17]. A typical retrieval system would also incorporate different techniques, namely, query enhancement [13], visual similarity search [16], and temporal search [16]. We previously reviewed the SIB image data manually to determine the feasibility and acceptability of wearable cameras to assess self-care in people living with HF [21]. Other studies have also manually reviewed camera images [18-20], which is time-consuming and laborious. In this study, we used the E-Myscéal system [22] to rapidly retrieve and review images of relevance. The E-Myscéal system eliminated the need for manual image review, enabled cross-media querying, supported temporal queries, allowed data filtering and clustering, and provided fast retrieval of the most relevant images.

E-Myscéal was the top-ranked retrieval system at the LSC’22 challenge [28], achieving the highest number of relevant items in the shortest time, and represents the current state-of-the-art in lifelog inquiry and retrieval systems. E-Myscéal exhibits a heightened sensitivity toward certain search terms. For instance, the search term “taking medication” generated considerably higher scores compared to “medication intake,” leading to a significant difference in the number of images retrieved. Hence, the user needs to consider what search terms will work well for any category or daily activities. The flexibility of the E-Myscéal system allowed a wide range of search terms to be tested and evaluated for any category, which the user needs to consider in future research.

Using the E-Myscéal system, the most frequently observed activities were eating, drinking, and screen time. These findings suggest that people living with HF are sedentary and spend considerable time in the presence of some form of screen (eg, television and computer). However, it was not possible to determine from the images whether the person was watching the television. As highlighted above, the Narrative Clip used in this study captured images every 30 seconds and captured the presence of objects in front of the person (eg, television, book, or dinner plate), as well as the context (eg, sitting at a table, being inside or outside, or seated in a car); however, unless the camera captured the person lifting a cup to their mouth it was difficult to determine whether they actually performed the behavior. Thus, while the technology offers a potential for capturing potential activities, a process for confirming what the person was doing is needed. In previous studies [18-20], wearable cameras have been used to support traditional self-report methods or to provide a primary record of dietary intake, however, no studies have used wearable cameras as the primary method of data collection; future research is needed to address this.

If the recorded images do represent a fair reflection of what people with HF were doing throughout the day, then they highlight some interesting patterns. For example, excessive screen or sedentary time might highlight people’s preferences (eg, sitting and watching television) [30-32] or could indicate fatigue, a common symptom of HF. Further, the high number of recorded images of eating and drinking could give some indication of what people with HF were eating (eg, foods high in salt) [33], and the frequency of drinking fluids, which may be an issue for people with HF who are on fluid restriction [4,33]. Despite the importance of taking prescribed medications and engaging in physical activities such as gardening and shopping, the number of images recorded by wearable cameras for these activities was low. The lower number of medication-taking images may have resulted from people not wearing their cameras first thing in the morning or later in the evening when medications were taken. However, it is more likely that the sensitivity of E-Myscéal for detecting medication taking was lower than for other ADLs used in this study. In terms of physical activities, it is plausible that participants removed the camera when shopping or doing physical activity. However, this is unlikely. In the original SIB study, adherence to wearing the camera was high [26]. Moreover, previous research has shown that people wear these types of wearable cameras when undertaking physical activity and they are useful for providing contextual information on activity and sedentary behavior [34,35].

We also found that most images of ADLs were observed before midday. Further, 1 possible reason is that participants did not wear the camera in the afternoon—they may have removed the camera or forgotten [36] to put it back on, which would result in a lower number of images captured during this time. Another possibility is that the camera battery might not have been fully charged during the previous night, which resulted in the device turning off [37] in the afternoon and fewer images being captured. Additionally, it is possible that participants were spending their afternoons sleeping by taking naps or resting [38-40], which would result in fewer activities [41] being captured during this time. Lastly, issues with the wearable camera time set-up, which may have led to inaccurate time stamps on the images captured, could also have contributed to the observed differences in activity patterns.

Furthermore, there were observed changes in the participants’ daily activities both before hospital admission and after discharge, which could be attributed to the time participants wore the device or differences in performed ADLs. If the latter, these findings suggest that participants in specific ADLs may have been influenced by their health condition or recovery process. For instance, if they were less active before hospitalization or had mobility limitations, it could result in fewer captured images during that period. The increase in medication images after discharge suggests a heightened focus on medication management and adherence during recovery. Conversely, the decrease in screen time images implies a shift in attention or engagement with electronic devices, possibly due to increased social interaction or involvement in other activities. These findings provide insights into potential changes in participants’ daily routines and behaviors, but further analysis, interpretation, and context are needed to fully understand the reasons behind these variations and their impact on overall well-being.

The temporal differences in the number of images recorded might have implications for managing HF in older adults, as health care professionals could use this information to optimize future self-care strategies such as designing customized interventions to promote medication taking, physical activity, minimizing sedentary behavior, and promoting a healthy diet [4,33]. The use of wearable cameras to augment self-care interventions was highlighted in a previous scoping review [42]. In that review, the authors suggested that people with a new diagnosis of HF could wear a camera for several days. On their return to an outpatient clinic, a nurse specialist or other health professional could review images alongside the individual to identify specific activities and use that as an opportunity to question them about self-care practices and offer tailored suggestions for improvement. The E-Myscéal platform would permit such rapid review.

A strength of this study was the use of wearable cameras to record first-person perspectives of real-life experiences and associated contexts for people living with HF. This approach provides a rich source of data to better understand people’s lived experiences and context for self-management and could be used to enhance patient outcomes by enabling health care providers to access more personalized and precise information about a person’s condition, which could ultimately lead to better care and treatment. This study is also the first to use the E-Myscéal system, a flexible and efficient solution for processing large volumes of images in a short time, addressing image preprocessing and classification challenges. However, a limitation of this approach is the sensitivity of E-Myscéal to specific search terms, which may influence the number and types of images retrieved, which could affect interpretation. It is important to consider these factors when interpreting this study’s findings and drawing conclusions about the daily activity patterns of individuals living with HF. Future research is needed to investigate the reasons behind these differences and develop strategies to improve the accuracy and reliability of wearable camera data collection.

### Conclusions

Wearable cameras are a valuable tool for understanding daily activities and self-management in people living with HF. E-Myscéal efficiently retrieves images, emphasizing the need for careful search term selection. These findings suggest a potential for tailored HF interventions based on temporal activity patterns, despite challenges in confirming specific behaviors from images. Further research is needed to address observed activity variations and enhance data accuracy.

## Appendix

Multimedia Appendix 1 The method by which E-Mysceal determines the number of images for a given query.

## Abbreviations

ADL: activity of daily living
HF: heart failure
LSC’22: Lifelog Search Challenge
SIB: Seeing is Believing

## Data Availability

The data sets generated and analyzed during this study are not publicly available as the camera images include identifiable information. However, other data are available from the corresponding author on reasonable request.
