# Supplementary material for: Daily Activity Lifelogs of People With Heart Failure: Observational Study
Source: JMIR Form Res. 2024 Feb 21;8:e51248. doi: 10.2196/51248 (PMC10918541; doi:10.2196/51248)
Supplement: Multimedia Appendix 1 [file formative_v8i1e51248_app1.docx]

# Multimedia Appendix 1

THIS TEXT BELOW REFERS TO THE METHODOLOGY THROUGH WHICH THE NUMBER OF IMAGES DISPLAYED BY E-MYSCEAL FOR ANY GIVEN QUERY IS DECIDED.

To find a suitable cut-off point for images, we used the Mean Thresholding method that relies on mean and standard deviation. In other words, for each search query, we measured the similarity scores of all images and set the threshold as:

$$\theta=\mu+k\sigma$$

whereas $\mu$ is the mean, $\sigma$is the standard deviation of the similarity scores, and $k$ is user-defined. To find the best value of k, we created a small validation set with one-day worth of data from one user and nine topics: ‘Reading’, ‘Doing Sudoku’, ‘Eating’, ‘Using Phone’, ‘Laundry’, ‘Watching TV’, ‘Drinking’, ‘Cooking’. We analysed precision, recall, and F1 scores using different values of $k$. We also observed the differences in number of images between the ground truth and what the system returns. The results are illustrated in Supplemental Figure 1. There is an obvious trade-off between recall and precision and F1 score tends to increase as $k$ approaches [2.0, 2.2]. Looking at the size differences, the system would return the best number of images when $k=2.0$ . Thus, we decided to set $k=2.0$ for the rest of the study.


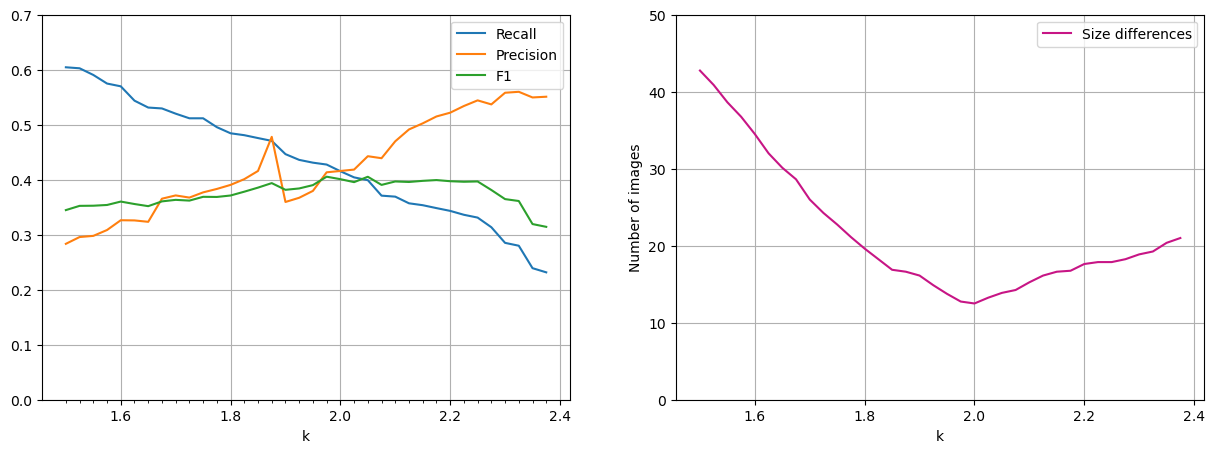


**Figure S1.** Recall, precision, F1 scores, and size differences to ground truth over different values of k.
